# Progressive hemorrhagic injury and ischemia after severe traumatic brain injury according to hemoglobin transfusion thresholds: a post-hoc analysis of the transfusion requirements after head trauma trial

**DOI:** 10.1186/s13054-024-04981-5

**Published:** 2024-07-03

**Authors:** André L. N. Gobatto, Davi Solla, Sérgio Brasil, Fabio S. Taccone, Carlos G. Carlotti Jr, Luiz Marcelo S. Malbouisson, Wellingson S. Paiva

**Affiliations:** 1 Division of Neurosurgery, Department of Neurology, School of Medicine, University of São Paulo, Av. Dr. Eneas de Carvalho Aguiar 255, São Paulo, Brazil; 2 Surgical Intensive Care Unit, Anesthesiology Division, Hospital das Clínicas, University of São Paulo Medical School, São Paulo, Brazil; 3 Department of Intensive Care, Hôpital Universitaire de Bruxelles (HUB), Université Libre de Bruxelles (ULB), Brussels, Belgium

*Trial registration*: ClinicalTrials.gov, NCT02203292. Registered on 29 July 2014.

Following severe traumatic brain injury (TBI), up to 50% of patients develop significant anemia, which compromises oxygen delivery to the cerebral tissue [1]. Furthermore, about 20–50% of TBI cases complicate with progressive hemorrhagic injuries (PHI) within the first 72 h post-injury [2] and 19–68% develop vasospasm [3]. Such combination of events increase the risks for cerebral ischemia and suggest blood transfusion as a strategy to mitigate the possibility of additional tissue hypoxia. Nevertheless, defining the optimal hemoglobin thresholds to trigger transfusion and prevent additional hypoxic damages in TBI patients remains elusive. Therefore, the purpose of the present study was to assess whether different transfusion strategies can impact hemorrhagic and ischemic outcomes in TBI.

This study presents a post hoc analysis of the Transfusion Requirements After Head Trauma (TRAHT) trial, an open-label, parallel, feasibility, randomized controlled trial investigating two red blood cell transfusion strategies: a "restrictive" approach (hemoglobin < 7 g/dL) versus a "liberal" approach (hemoglobin < 9 g/dL) [4]. Inclusion and exclusion criteria have been previously outlined [4]. Patients adhered to their assigned transfusion strategy for up to 14 days or until death or ICU discharge, whichever occurred first. All patients were monitored using serial transcranial Doppler ultrasound (TCD) examinations. TCD provides cerebral blood velocities (CBv) as surrogate measures for cerebral blood flow, with multiple applications in critical care [5]. Cerebral vasospasm was defined using Lindegaard and Soustiel indexes, based on cerebral (middle cerebral [MCA] and basilar) and extracranial (internal carotid and vertebral) arteries blood velocities [5]. All patients were submitted to brain CT at admission following the discretion of the attending teams. The primary endpoint was observing the occurrence of new ischemic events post-randomization. Also, we described the occurrence of PHI, which was defined as the appearance of new or enlarged intracerebral hematomas by a minimum of 10% its previous volume, enlargement of subdural or epidural hematomas, or unexpected post-surgical hematomas at the operative site, with no minimum volume threshold. Extensive ischemia corresponded to a hypodense area suggestive of vascular etiology on CT scans, involving more than two segments of a cerebral artery, as MCA M3 and M4 per example. All radiological evaluations were analyzed by an independent radiologist blinded to the study allocation. For descriptive purposes, categorical variables were presented through relative and absolute frequencies and compared by means of the chi-squared or Fisher exact test, as appropriate. All tests were two-sided and final p values under 0.05 were considered statistically significant. The post-hoc analysis was considered exploratory and no multiple tests correction was implemented.

Between August 2014 and June 2016, 44 patients were included in the final analysis; of these, 23 were allocated to the restrictive group and 21 to the liberal group. Mean age was 35 ± 13 years and a median admission Glasgow Coma Scale (GCS) score was 4 (3–7). The overall sample characteristics are shown in Supplementary Table whilst our main results are shown in Table 1. Pre and post-randomization head CT scans were performed in 38 out of 44 patients (86%), whilst pre and post-randomization TCD scans were performed in the entire sample for vasospasm. PHI was detected 3 patients (16.7%) in the restrictive group and 1 patient (5.0%) in the liberal group (*p* = 0.35). New ischemic events were identified in 3 patients (16.7%) in the restrictive group and in 4 patients (20.0%) in the liberal group and (*p* = 0.98). Most ischemic events in the liberal group were new injuries (3/4), whereas patients in the restrictive group showed worsening of previous ischemia alongside new infarcts. Such complications as PHI or new ischemic events were not observed in the same patients. Extensive ischemia was exclusively observed in the restrictive group (*p* = 0.029). Pre-randomization ischemia and vasospasm were present in 43% and 10% of patients in the liberal strategy group, respectively, compared to 70% and 22% in the restrictive group (*p* = 0.127 and *p* = 0.416, respectively). Ischemic events were more frequent among those patients with traumatic subarachnoid hemorrhage on admission and those with pre-randomization cerebral vasospasm. Although more prevalent in the restrictive group, vasospasm severity was not significantly different between groups. A negative correlation was observed between hemoglobin concentration and mean MCA blood velocities (*r* =  − 0.265, *p* < 0.01).

**Table 1:** Main results

Variable	Group		p value
	Liberal (21)	Restrictive (23)	
Tomographic follow-up according to the allocated group			
Post-randomization head CT	19/21 (90)	19/23 (83)	0.66
Post-randomization PHI	1 (5)	3 (16)	0.35
Post-randomization ischemia	4 (21)	3 (16)	> 0.9
Worsening of previous ischemia	1/4 (25)	3/3 (100)	0.14
New ischemia	3/4 (75)	2/3 (67)	> 0.9
Extensive ischemia	0/4 (0.0)	3/3 (100)	0.02
Pre-randomization ischemia	9 (43)	16 (70)	0.12
Vasospasm prevalence	4 (19)	15 (65)	< 0.01
Lindegaard ratio (among patients with vasospasm)	4.2 ± 0.8	4.4 ± 1.2	0.09
MCA blood velocities (cm/s)	Pre-rand. 112.4 ± 21.6	Pre-rand. 109.8 ± 15.9	< 0.02
	Post-rand. 71.4 ± 18.2	Post-rand. 101.2 ± 16.7	
Tomographic follow-up according to tSAH			
Post-rand. new or ischemic zone enlargement n = 7	Pre-rand. tSAH 6 (85)	No pre-rand. tSAH 1 (14)	0.01

Data presented as n (%). CT, computed tomography; PHI, progressive hemorrhagic injury; TCD, transcranial Doppler; tSAH, traumatic subarachnoid hemorrhage

In this analysis, we observed higher cerebral blood velocities among patients randomized to a restrictive transfusion approach, as well as vasospasm prevalence and extensive ischemic injuries in this group. However, the occurrence of PHI and new ischemic events was similar between groups. Although causal correlations were precluded given our limitations, this study suggest an association between anemia and vasospasm. Further research to establish evidence-based guidelines for blood transfusions in TBI are warranted.

### Supplementary Information

Additional file 1.

## Data Availability

The dataset is available upon reasonable request.

## Abbreviations

GCS: Glasgow Coma Scale
GOS: Glasgow outcome Scale
ICP: Intracranial pressure
ICU: Intensive care unit
RBC: Red blood cells
SPSS: Statistical package for the social sciences
TBI: Traumatic brain injury
TCD: Transcranial Doppler
TRAHT: Transfusion requirements after head trauma
