# Supplementary material for: Progressive hemorrhagic injury and ischemia after severe traumatic brain injury according to hemoglobin transfusion thresholds: a post-hoc analysis of the transfusion requirements after head trauma trial
Source: Crit Care. 2024 Jul 3;28:218. doi: 10.1186/s13054-024-04981-5 (PMC11223415; doi:10.1186/s13054-024-04981-5)
Supplement: Supplementary file 1 — Additional file 1. [file 13054_2024_4981_MOESM1_ESM.docx]

| **Sup. TABLE. Sample characterization according to the allocated group** | | | |  |  |
| --- | --- | --- | --- | --- | --- |
| **Variable** |  | **Group** | | | **p value** |
|  | **Total (44)** | **Liberal (21)** | **Restrictive (23)** | |  |
| Age, years | 35 ± 13 | 33 ± 11 | 36 ± 15 | | 0.34 |
| Male | 40 (81) | 20 (95) | 20 (87) | | 0.61 |
| GCS ate hospital admission | 4 [3-7] | 4 [3-7] | 5 [3-7] | | 0.90 |
| Pupil alteration (one or both) | 20 (46) | 7 (33) | 13 (59) | | 0.09 |
| Patients transfused RBC before randomization | 24 (55) | 9 (43) | 15 (65) | | 0.14 |
| RBC units per patient transfused before randomization | 4.4 ± 6.2 | 3.1 ± 5.2 | 5.6 ± 6,9 | | 0.24 |
| Patients transfused other blood products before randomization | 4 (10) | 0 | 4 (17) | | 0.11 |
| Head CT |  |  |  | |  |
| Compressed cisterns | 35 (81) | 17 (85) | 18 (78) | | 0.70 |
| Midline deviation > 5 mm | 26 (59) | 10 (48) | 16 (70) | | 0.14 |
| Diffuse axonal lesion | 14 (33) | 9 (45) | 5 (22) | | 0.10 |
| Subarachnoid hemorrhage | 22 (50) | 13 (62) | 9 (39) | | 0.13 |
| Intracranial hematoma | 35 (80) | 17 (81) | 18 (78) | | 0.82 |
| Neurosurgical intervention |  |  |  | |  |
| None | 11 (25) | 5 (24) | 6 (26) | | 0.86 |
| Epidural hematoma drainage | 6 (14) | 4 (19) | 2 (9) | | 0.40 |
| Subdural hematoma drainage | 5 (11) | 1 (5) | 4 (17) | | 0.35 |
| Intracerebral hemorrhage drainage | 2 (5) | 1 (5) | 1 (4) | | 1.00 |
| Decompressive craniectomy | 17 (39) | 7 (33) | 10 (44) | | 0.49 |
| Other | 5 (11) | 3 (14) | 2 (9) | | 0.66 |
| Traumatic associated injuries | 31 (70) | 16 (76) | 15 (65) | | 0.43 |
| Prognostic scores |  |  |  | |  |
| SAPS3 at ICU admission | 56 ± 13 | 55 ± 12 | 57 ± 12 | | 0.49 |
| CRASH 14-day death risk | 40 ± 18 | 39 ± 18 | 41 ± 19 | | 0.82 |
| CRASH 6-month unfavorable outcome risk | 70 ± 19 | 70 ± 17 | 70 ± 22 | | 0.91 |
| Injury severity score | 29 ± 9 | 28 ± 9 | 31 ± 9 | | 0.40 |
| IMPACT | 49 (15) | 47 (17) | 52 (14) | | 0.29 |
| Injury-to-randomization, hours | 71 ± 38 | 75 ± 41 | 65 ± 34 | | 0.35 |
| Data presented as n (%), mean ± standard deviation or median [quartiles]. | | | | | |
